# Routine Administration of a Multispecies Probiotic Containing *Bifidobacterium* and *Lactobacillus* to Very Low Birth Weight Infants Had No Significant Impact on the Incidence of Necrotizing Enterocolitis

**DOI:** 10.3389/fped.2021.757299

**Published:** 2021-10-28

**Authors:** Brian A. Juber, Timothy J. Boly, Graeme J. Pitcher, Steven J. McElroy

**Affiliations:** ^1^Department of Pediatrics, University of Nebraska Medical Center, Omaha, NE, United States; ^2^Stead Family Department of Pediatrics, University of Iowa, Iowa City, IA, United States; ^3^Division of Pediatric Surgery, Department of Surgery, University of Iowa, Iowa City, IA, United States; ^4^Department Pediatrics, University of California, Davis, Davis, CA, United States

**Keywords:** necrotizing enterocolitis (NEC), bifidobacteria, probiotics, VLBW (very low birth weight), ELBW (extreme low birth weight infants)

## Abstract

**Background:** Necrotizing enterocolitis (NEC) is the leading cause of gastrointestinal morbidity in preterm infants, and prevention and treatment strategies have remained largely unchanged over the past several decades. As understanding of the microbiome has increased, probiotics have been hypothesized as a possible strategy for decreasing rates of NEC, and several studies have noted significant decreases in rates of NEC after initiation of probiotics in preterm infants. However, a recent AAP report cited caution on the use of probiotic use in part because studies of probiotic use in ELBW infants are lacking. As our unit began routine use of probiotics for all infants <33 weeks in 2015 and we are a leading institution for intact survival of ELBW infants, we attempted to answer if probiotic use can impact the rate of NEC in VLBW and ELBW infants.

**Methods:** We conducted a single-center retrospective chart review of infants with modified Bell's stage ≥2a NEC for the 4 years prior to and 5 years after initiation of a protocol involving routine supplementation of a multispecies probiotic to premature infants at the University of Iowa, Stead Family Children's Hospital. The primary outcome measures were rates of modified Bell's stage ≥2a NEC and all-cause pre-discharge mortality at our institution before and after initiation of routine probiotic supplementation in 2015.

**Results:** In our institution, neither the rates of modified Bell's stage ≥2a NEC, nor the rates of all-cause mortality were significantly altered in very low birth weight (VLBW) infants by the initiation of routine probiotic use (NEC rates pre-probiotic 2.1% vs. post-probiotic 1.5%; all-cause mortality rates pre-probiotic 8.4% vs. post-probiotic 7.4%). Characteristics of our two cohorts were overall similar except for a significantly lower 5-minute APGAR score in infants in the post-probiotic epoch (pre-probiotic 8 vs. post-probiotic 6 *p* = 0.0316), and significantly more infants in the post-probiotic epoch received probiotics (pre-probiotics 0% vs. post-probiotics 65%; *p* < 0.0001). Similarly, probiotic use had no impact on the incidence of NEC when we restricted our data to only extremely low birth weight (ELBW) infants (pre-probiotics 1.6% vs post-probiotics 4.1%). When we restricted our analysis to only inborn infants, probiotics still had no impact on NEC rates in VLBW infants (1.5% pre- and 1.1% post-probiotic, *p* = 0.61) or ELBW infants (2% pre- and 2.1% post-probiotic, *p* = 0.99)

**Conclusions:** Contrary to other studies, we found no significant difference in rates of modified Bell's stage ≥2a NEC or all-cause pre-discharge mortality in VLBW infants following routine administration of a multispecies probiotic supplement.

## Introduction

Necrotizing enterocolitis (NEC) is the leading cause of gastrointestinal morbidity and mortality in premature infants, with ~6–9% of very low birth weight (VLBW) infants developing NEC (1–3). Mortality rates are typically 20–30% and can be as high as 40% in the extremely low birth weight (ELBW) population of infants (4). Despite intensive research into pathophysiology and novel therapeutics, medical therapies have largely remained the same over the past several decades, consisting of nasogastric drainage, bowel rest, broad-spectrum intravenous antibiotics, and parenteral fluids and nutrition (5). In severe cases, surgery may be required for perforation or signs of bowel necrosis, but the optimal modality of surgical management remains unclear (6, 7).

Both the etiology and pathophysiology of NEC are diverse and multifactorial. Recently, microbiome studies in preterm infants have yielded valuable insights into its contributions to many disease processes, including NEC. The human microbiome consists of 10–100 trillion symbiotic bacteria, most of which are harbored in the gastrointestinal tract (8). The establishment and composition of the neonatal microbiome, especially in preterm infants, is adversely affected by many prenatal and postnatal factors, including mode of delivery, breast milk vs. formula feeding, and both duration and type of antibiotic exposure (9–14). Distinct differences in microbiome composition have been noted in premature infants compared to term infants (15). These alterations are especially pronounced in premature infants with NEC, implicating a perturbed and non-diverse microbiome as a risk factor for later development of NEC (3, 16–19).

Probiotics are defined as live microorganisms which when administered in adequate amounts confer a health benefit on the host, and they are often used to diversify the microbiome in both the pediatric and adult populations (20). Multiple studies and meta-analyses have demonstrated that administration of probiotics to preterm infants in the neonatal intensive care unit (NICU) is likely safe and may decrease the risk of NEC and mortality (21–26). However, despite widespread use in other developed countries, the practice of administering probiotics to preterm infants remains relatively limited and controversial in the United States (27). A recent survey of NICUs in the United States found that despite a lack of evidence-based guidelines, ~1 in 10 NICUs give VLBW infants probiotic supplements either routinely or to select infants as a part of their clinical practice (28). Part of this controversy stems from the fact that probiotics are considered a dietary supplement by the United States Food & Drug Administration (FDA), meaning that probiotics are not required to meet the same stringent regulatory standards as medications that are marketed as a drug to treat a certain disorder. Consequently, there is marked heterogeneity in commercially available probiotic products. Since the effects of probiotics are highly strain specific (29), it is difficult to compile aggregate data from different prospective studies and clinical trials. Moreover, it has also been noted that the contents of commercially available probiotic preparations often do not match what is listed on the label (30). Given the lack of definitive evidence for efficacy, conflicting data on safety, and potential for harm in a highly vulnerable population of patients, a recently published American Academy of Pediatrics (AAP) clinical report was unable to support the routine, universal use of probiotics in preterm infants, particularly ELBW infants (31).

Multiple formulations of probiotics exist, with *Bifidobacterium* and *Lactobacillus* as the most common strains used (32). *Bifidobacterium* is a genus of gram-positive anaerobic bacteria that was first isolated from the stool of breastfed infants in 1899, and it has been associated with a reduction in NEC in preterm infants (33, 34). Similarly, *Lactobacillus* is a genus of gram positive facultative anaerobic bacteria which has also been demonstrated to have multiple positive microbiological and immunological effects on the host (35). Each genus confers specific benefits, but recently published population-based research suggests that a combination of *Bifidobacterium* and *Lactobacillus* is safe and bestows the most benefit to the neonatal population (26). An additional meta-analysis published several years earlier also demonstrated there was a highly significant reduced risk of NEC and mortality when a multiple strain probiotic was used, which was not present with use of single strain probiotic formulations (24).

In 2015, our institution introduced a protocol for routine probiotic supplementation in premature infants. This decision was supported by previously published studies demonstrating a significant decrease in NEC with the multispecies probiotic consisting of four *Bifidobacterium* species and *Lactobacillus rhamnosus* (22, 23). However, there is currently a paucity of literature examining probiotic use in ELBW infants. Because of our institution's high rate of neurodevelopmentally intact survival for VLBW and ELBW infants (36) as well as our anecdotally low baseline rate of NEC (2–4%), we performed a retrospective cohort study examining rates of NEC in VLBW infants before and after initiation of routine supplementation of a multispecies probiotic. We also performed a subgroup analysis of rates of NEC in ELBW infants born <1,000 grams. We hypothesized that we would see lower rates of NEC and all-cause mortality in the post-probiotic epoch compared to the pre-probiotic epoch in both the VLBW and ELBW cohorts.

## Methods

### Our Context, Study Design and Selection of Study Population

The Stead Family Children's Hospital at the University of Iowa has an 87-bed level 4 NICU that has all subspecialty medical and surgical services and acts as both an inborn birth center and a referral center for Iowa and the surrounding states. The NICU admits an average of 153 VLBW and 71 ELBW infants per year. Our unit is known for its traditionally low NEC rate and its high rates of intact survival at the limits of viability (36). This was a single-center retrospective cohort study primarily focusing on preterm VLBW infants for the 4 years preceding and 5 years following implementation of a protocol for routine supplementation of a multispecies probiotic. The study was approved by the University of Iowa Institutional Review Board (IRB#201410743).

To develop our dataset, all infants with pediatric surgery consults were identified in the 4 years preceding and 5 years following the initiation of probiotics at our institution. Per unit protocol, all infants who have concern for NEC (medical or surgical) have a pediatric surgical consult placed. Infants were excluded due to major congenital abnormalities (congenital diaphragmatic hernia [CDH], gastroschisis, or omphalocele), anatomic obstruction of the gastrointestinal tract (intestinal atresia, malrotation, or Hirschsprung disease), inguinal hernia repair, G-tube placement, or peritoneal dialysis catheter placement.

### Data Collection and Identification of Infants With NEC

Infants with pediatric surgical consults during the years 2011–2019 who did not have the above exclusion criteria had data collected regarding baseline characteristics, feeding practices, risk factors for intestinal illness, and markers of illness severity. Chart review and data collection was conducted by two individuals who were also part of the consensus group. Study data were collected and managed using REDCap (Research Electronic Data Capture) electronic data capture tools at the University of Iowa (37, 38).

Within this cohort, a diagnosis of modified Bell's stage ≥2a NEC, spontaneous intestinal perforation (SIP), or neither condition was determined by consensus of a group consisting of pediatric surgeons and neonatologists. Consensus on a diagnosis was defined as agreement of at least 3 out of the 4 group members. We used the modified Bell's classification stage ≥2a to classify cases of NEC (39). Infants who did not meet these criteria in general had a single episode of bilious emesis with a negative workup, or had a concern for NEC (i.e., bloody stools, abdominal distention, abdominal discoloration, or increasing gastric residuals) but did not meet consensus criteria for Bell's stage ≥2a NEC. We then separated infants into the pre-probiotic and post-probiotic cohorts based on date of birth. Because routine probiotic supplementation began in 2015, 2011–2014 was defined as the pre-probiotic epoch and 2015–2019 was defined as the post-probiotic epoch.

### Probiotic Protocol

Beginning in 2015, our institution began routine administration of Ultimate Flora Baby Probiotic^®^ (www.renewlife.com), a multispecies probiotic formulation consisting of four *Bifidobacterium* species (*Bifidobacterium breve, bifidum, infantis*, and *longum*) plus *Lactobacillus rhamnosus* GG at a concentration of 2 × 10^9^ colony forming units per 0.5 g. The probiotic was prepared daily by our pharmacy under conditions that minimize the risk of contamination by adding 0.5 g of probiotic to 2.5 mL of 2.5% dextrose in sterile water. This formulation is then administered to eligible infants via enteral gavage once daily prior to an enteral feed. Infants eligible for probiotic supplementation were at least 3 days old, born at <33 0/7 weeks gestational age, with a corrected post-menstrual age (PMA) of at least 24 0/7 weeks, who were also receiving intake of at least 6 mL of enteral feedings per day. The probiotic is routinely held during periods of NPO status and is otherwise continued daily until the infant reaches a corrected PMA of 36 0/7 weeks, at which point it is discontinued.

### Statistical Analysis

Parametric *t*-testing was performed for continuous variables and chi-square testing was performed for categorical variables as appropriate using GraphPad Prism v9. Statistical significance was defined as a *p* < 0.05, a non-significant trend was defined as a *p*-value more than 0.05 but <0.1, and a non-significant (NS) finding was defined as a *p* > 0.1. All error values reported are standard error of the mean.

## Results

### Identification of Infants With NEC

**Figure 1:**
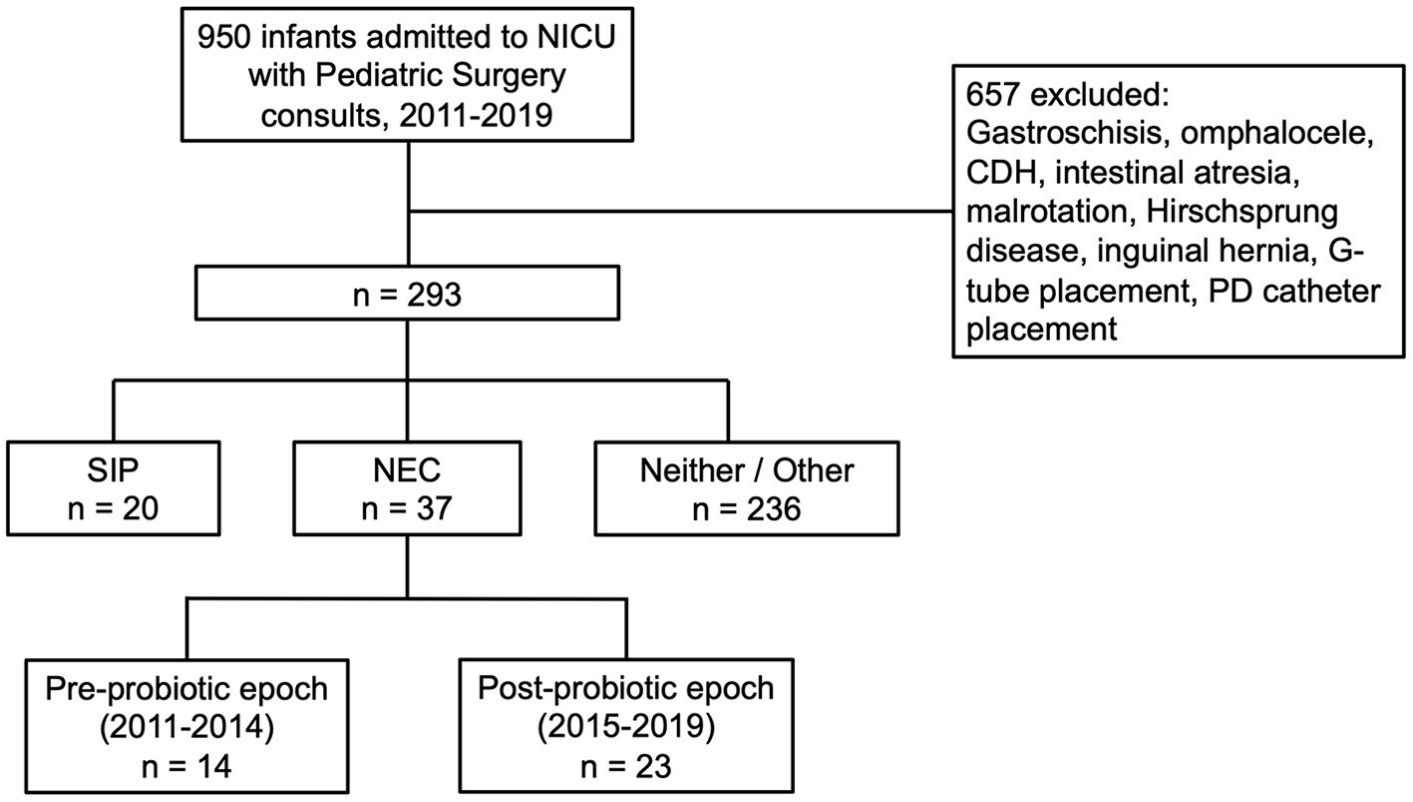
Flow diagram of patients included in the study. From an original sample of 950 pediatric surgery consults from 2011 to 2019, through consensus of a group of neonatologists and pediatric surgeons, we identified 14 cases of NEC in the pre-probiotic epoch (2011–2014) and 23 cases of NEC in the post-probiotic epoch (2015–2019).

A total of 950 infants were identified with pediatric surgical consults between the years of 2011–2019 (Figure 1). Of those, 657 were excluded from analysis due to the presence of major congenital abnormalities (CDH, gastroschisis, or omphalocele), anatomic obstruction of the gastrointestinal tract (intestinal atresia, malrotation, or Hirschsprung disease), or consult only for inguinal hernia repair or gastrostomy tube or peritoneal dialysis catheter placement, leaving a total of 293 infants remaining for data extraction from electronic medical records. Following data extraction, consensus was obtained from a group of neonatologists and pediatric surgeons on cases of modified Bell's stage ≥2a NEC, resulting in a total of 37 cases of NEC between 2011 and 2019. There were 14 cases of NEC in the pre-probiotic epoch (2011 and 2014) and 23 cases of NEC in the post-probiotic epoch (2015 and 2019). The other 256 infants in the set were identified as having a SIP (20 infants) or having some other diagnosis (236 infants).

### Baseline Characteristics of Mothers and Infants

Data were collected regarding gestational age, birth weight, sex, race, birth location, maternal parity, prolonged rupture of membranes (more than 18 h), antenatal betamethasone administration (at least 1 dose), mode of delivery (vaginal birth vs. Cesarean section), small for gestational age (SGA, defined as birth weight <10th percentile on Fenton growth curve), maternal Medicaid status (as a marker for maternal socioeconomic status), and receipt of antibiotics immediately upon NICU admission for each case of NEC in the pre-probiotic and post-probiotic epochs (Table 1). Maternal characteristics were similar between the two groups with no significant differences. Infant characteristics were overall similar between both epochs. There were no significant differences in gestational age, birth weight, sex, race, SGA status, or rate of antibiotic use amongst the infants. We did find that infants with NEC in the post-probiotic epoch had a significantly lower 5 min APGAR score compared to infants with NEC in the pre-probiotic epoch (mean APGAR score 6.3 vs. 7.8; *p* = 0.0316).

**Table 1:** Baseline characteristics of infants diagnosed with NEC and their mothers before and after initiation of probiotics in 2015.

	**Pre-probiotic epoch (2011–2015) *n* = 14**	**Post-probiotic epoch (2015–2020) *n* = 23**	**Significance**
Gestational age	30.6 (±1.2)	28.7 (±1.1)	NS
Birth weight (g)	1,628 (±240)	1,332 (±216)	NS
Sex			NS
Male (%)	7 (50)	15 (65)	
Female (%)	7 (50)	8 (35)	
Race			NS
White (%)	10 (71)	15 (65)	
Black (%)	2 (14)	3 (13)	
Other (%)	2 (14)	5 (22)	
Birth location			*p* = 0.074
Inborn (%)	12 (86)	12 (52)	
Outborn (%)	2 (14)	11 (48)	
Maternal parity	2.3 (±0.6)	1.9 (±0.2)	NS
Rupture of membranes >18 h (%)	2 (14)	6 (26)	NS
Antenatal steroids, at least 1 dose (%)	11 (79)	19 (83)	NS
Mode of delivery			NS
Vaginal (%)	10 (71)	12 (52)	
Cesarean (%)	4 (29)	11 (48)	
5-min APGAR score	7.8 (±0.5)	6.3 (±0.4)	*p* = 0.0316
Small for gestational age (%)	0 (0)	4 (17)	NS
Maternal Medicaid status (%)	9 (64)	9 (39)	NS
Antibiotics started at birth (%)	13 (93)	22 (96)	NS

*Infants in the post-probiotic epoch had a significantly lower 5-min APGAR score compared to those in the pre-probiotic epoch. Otherwise, there were no statistically significant differences between the two epochs*.

### Feeding Practices

Data were collected regarding day of life (DOL) enteral feeds initiated, DOL feeds reached 40 mL/kg/day (feed volume in our unit when feeds are fortified), DOL feeds first fortified to 24 kcal/oz, feeding amount at time of fortification, type of feeding (exclusive breast milk, exclusive formula, or mix), amount of enteral intake on day of diagnosis and for 3 days prior to diagnosis, and average total fluid intake (parenteral + enteral) for 3 days prior to diagnosis and through day of diagnosis for each case of NEC in the pre-probiotic and post-probiotic epochs (Table 2). There were no significant differences in feeding practices between the two epochs.

**Table 2:** Practices for feeding and probiotic use for infants diagnosed with NEC before and after initiation of probiotic supplementation in 2015.

	**Pre-probiotic epoch (2011–2015) *n* = 14**	**Post-probiotic epoch (2015–2020) *n* = 23**	**Significance**
DOL feeds initiated	1.9 (±0.6)	1.8 (±0.7)	NS
DOL feeds reached 40 mL/kg/day	7.0 (±2.1)	11.6 (±3.5)	NS
Type of feeding			NS
Breast milk and/or donor milk (%)	7 (50)	13 (59)	
Breast milk + preterm formula (%)	6 (43)	8 (36)	
Preterm formula (%)	1 (7)	1 (5)	
DOL feeds fortified	8.1 (±1.9)	12.8 (±3.2)	NS
Feeding amount at fortification (mL/kg/day)	61.9 (±9.6)	56.2 (±10.0)	NS
Average enteral intake on day of diagnosis (mL/kg/day)	98.4 (±12.6)	85.4 (±14.0)	NS
Enteral intake 1 day prior to diagnosis (mL/kg/day)	94.3 (±12.0)	79.9 (±14.8)	NS
Enteral intake 2 days prior to diagnosis (mL/kg/day)	94.9 (±11.7)	77.7 (±15.3)	NS
Enteral intake 3 days prior to diagnosis (mL/kg/day)	86.8 (±11.7)	73.1 (±15.3)	NS
Average total fluid intake for 3 days prior to diagnosis (mL/kg/day)	129.4 (±3.0)	138.3 (±10.7)	NS
Probiotics administered (%)	0 (0)	15 (65)	*p* < 0.0001
DOL probiotics started	N/A	15.0 (±5.3)	N/A

*There were no significant differences in feeding practices in the post-probiotic epoch compared to the pre-probiotic epoch. Significantly more infants in the post-probiotic epoch received probiotics*.

### Probiotic Use

Data were collected regarding probiotic use and DOL probiotics were started (if applicable) for each case of NEC in the pre-probiotic and post-probiotic epochs (Table 2). Overall, within the cohort of NEC cases during the post-probiotic epoch, 15/23 (65%) infants received probiotics at some point in their course, more than the infants with NEC during the pre-probiotic cohort (0/14, 0%). Within the VLBW cohort in the post-probiotic epoch, 15/16 (94%) received probiotics, and 13/14 (93%) of ELBW infants received probiotics compared to 0% for both groups in the pre-probiotic epoch. Of the infants with NEC in the post-probiotic epoch who received probiotics, probiotics were started on average on DOL 15. To understand the impact of outborn birth, a secondary analysis done that was restricted to only infants who were inborn. In this secondary analysis, more infants in the post-probiotic epoch received probiotics compared to the pre-probiotic epoch (67% vs. 0%) and there was a near-significant delay of 3 weeks in probiotic initiation in outborn infants compared to inborn infants (probiotics initiated on DOL 28 vs. DOL 7, respectively; *p* = 0.0596).

### Rates of NEC for VLBW and ELBW Infants

**Figure 2:**
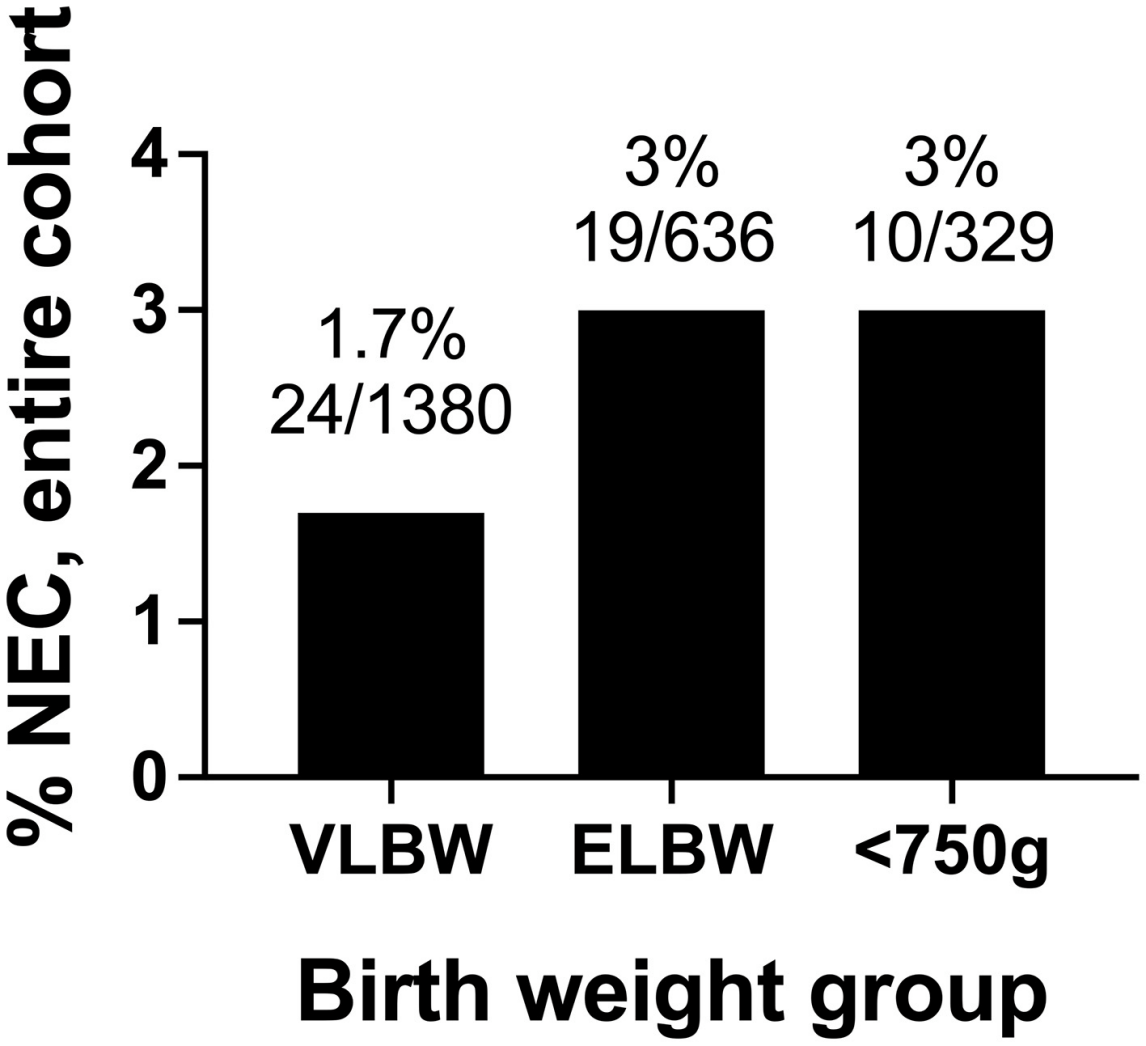
Rates of modified Bell's stage ≥2a NEC from 2011 to 2019 based stratified by birth weight. In our cohort, the incidence of NEC was 1.7% in VLBW infants, 3% in ELBW infants, and 3% in infants with birthweights <750 grams.

Between 2011 and 2019 at our institution, 1.7% of infants with birth weight <1,500 grams, 3.0% of infants with birth weight <1,000 grams, and 3.0% of infants with birth weight <750 grams developed modified Bell's stage ≥2a NEC (Figure 2). There were not statistically significant differences between the NEC rates based on weight.

**Figure 3:**
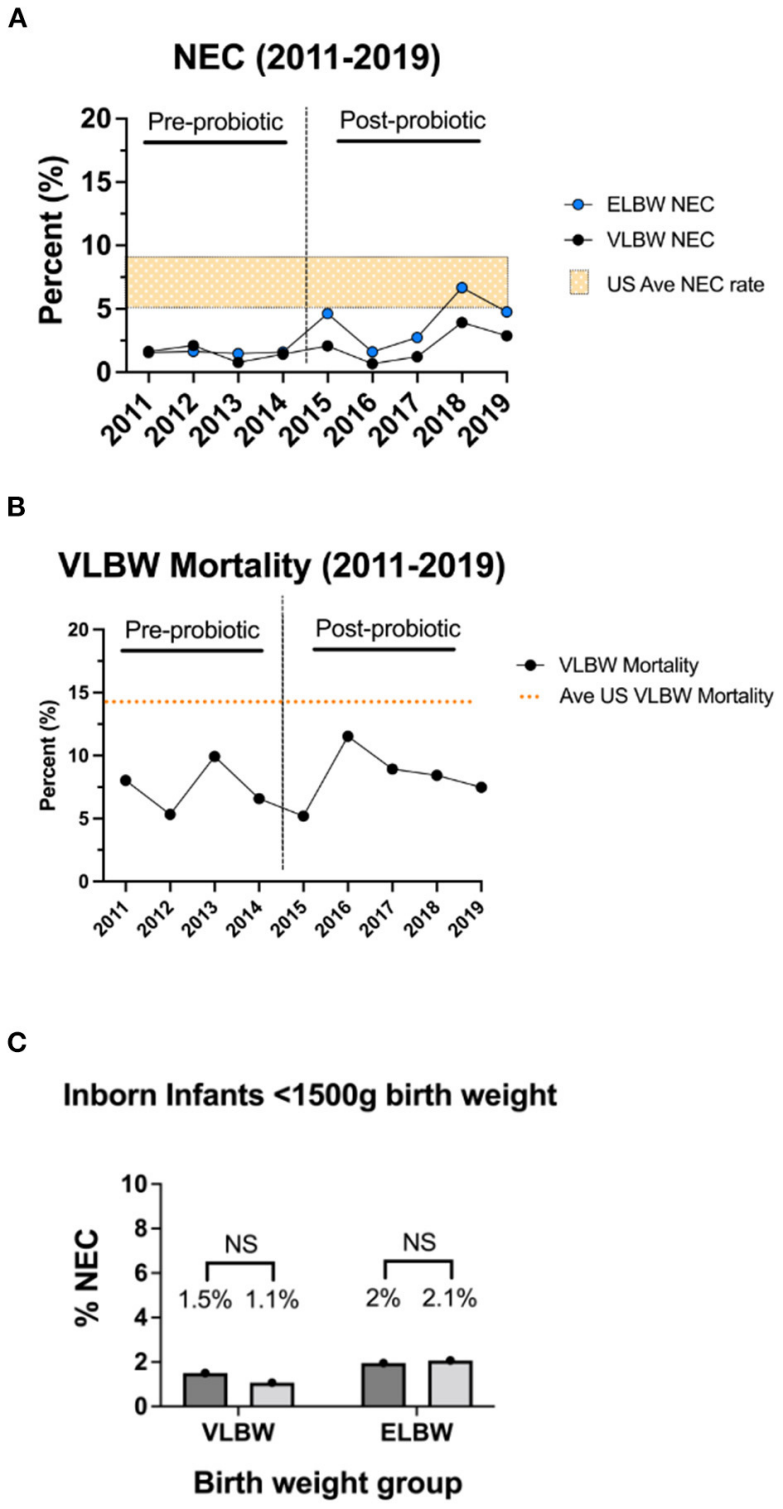
Rates of modified Bell's stage ≥2a NEC and all-cause mortality by year. Rates of modified Bell's stage ≥2a NEC **(A)** and all-cause pre-discharge mortality **(B)** for infants in the pre-probiotic and post-probiotic epochs. For the VLBW population, routine administration of probiotics was not associated with a significant difference in rates of NEC or all-cause mortality. Rates are compared to US average data denoted by orange bar (NEC range, **A**) or orange line (VLBW mortality rate, **B**) (3, 40). Sub-group NEC analysis of ELBW infants demonstrated a trend toward a higher rate of NEC in the post-probiotic epoch, but this did not reach statistical significance. **(C)** Rates of NEC in VLBW and ELBW inborn only infants pre- and post-probiotic initiation showing no difference between the inborn cohorts.

During the pre-probiotic epoch (2011 and 2014), 8 VLBW infants developed NEC, corresponding to an average NEC incidence of 1.5% (Table 3A, Figure 3A). Rates of NEC by year during the pre-probiotic epoch ranged from 0.8 to 2.1%. During the post-probiotic epoch (2015 and 2019), 16 VLBW infants developed Bell's stage ≥2 NEC, corresponding to an average NEC incidence of 2.1%. Rates of NEC by year during the post-probiotic epoch ranged from 0.7 to 3.9%. There was not a statistically significant difference in rates of NEC in the VLBW population between the pre-probiotic and post-probiotic epochs (Figure 3A). When a subgroup analysis was performed on only inborn VLBW infants, 8 infants developed NEC in the pre-probiotic epoch (1.5% average incidence with a yearly variance ranging from 0.8 to 2.1%.) and 8 infants developed NEC in the post-probiotic epoch (1.1% average incidence with a yearly variance ranging from 0.7 to 1.4%). These differences were not significantly different (Table 3B, Figure 3C).

**Table 3:** Incidence of Bell's stage ≥2 NEC and all-cause pre-discharge mortality for infants before and after initiation of probiotic supplementation in 2015.

	**Pre-probiotic epoch (2011–2014)**	**Post-probiotic epoch (2015–2019)**	**Significance**
**A: Inborn and outborn infants**			
NEC cases (Bell's stage 2+), VLBW <1,500 g (%; range)	8 (1.5; 0.8–2.1)	16 (2.1; 0.7–3.9)	NS
NEC cases (Bell's stage 2+), ELBW <1,000 g (%; range)	4 (1.6; 1.5–1.6)	14 (4.1; 1.6–6.7)	*p* = 0.090
All-cause pre-discharge mortality (%; range)	43 (7.4; 5.3–9.9)	67 (8.4; 5.2–11.5)	NS
**B: Inborn infants only**			
NEC cases (Bell's stage 2+), VLBW <1,500 g (%; range)	8 (1.5; 0.8–2.1)	8 (1.1; 0.7–1.4)	NS
NEC cases (Bell's stage 2+), ELBW <1,000 g (%; range)	5 (2.0; 1.5–3.2)	7 (2.1; 1.5–2.7)	NS

*Within the VLBW population, there were no significant differences in NEC or all-cause mortality (A). Upon a sub-group analysis of ELBW infants, a there was a trend toward a higher rate of Bell's >2a NEC in the post-probiotic epoch compared to the pre-probiotic epoch, but this did not reach statistical significance. There remained no statistically significant difference between the two epochs when only inborn infants were analyzed (B)*.

During the pre-probiotic epoch, 4 ELBW infants developed Bell's stage ≥2 NEC (1.6% average incidence with yearly variance from 1.5 to 1.6%), and 14 infants developed NEC in the post-probiotic epoch (4.1% incidence with yearly variance from 1.6 to 6.7%). These differences were not statistically different (Table 3A, Figure 3A). When a subgroup analysis was performed on only inborn ELBW infants, 5 infants developed Bell's stage ≥2 NEC in the pre-probiotic epoch (2.0% average incidence with a yearly variance ranging from 1.5 to 3.2%.) and 7 infants developed NEC in the post-probiotic epoch (2.1% average incidence with a yearly variance ranging from 1.5 to 2.7%). These differences were not significantly different (Table 3B, Figure 3C).

### Rates of All-Cause Mortality for VLBW Infants

During the pre-probiotic epoch, there were 43 VLBW deaths from all-cause mortality, corresponding to an average all-cause mortality incidence of 7.4%. VLBW mortality rates by year during the pre-probiotic epoch ranged from 5.3 to 9.9%. During the post-probiotic epoch, there were 67 VLBW deaths from all-cause mortality, corresponding to an average all-cause mortality incidence of 8.4%. VLBW mortality rates by year during the post-probiotic epoch ranged from 5.2 to 11.5%. There was no significant difference between VLBW mortality rates between the pre-probiotic and post-probiotic epochs (Table 3, Figure 3B).

### Risk Factors for Intestinal Illness

Data were collected regarding weight at diagnosis, DOL at diagnosis, PMA at diagnosis, clinical and culture positive early onset sepsis (EOS) and late onset sepsis (LOS), ratio of total stools to DOL of diagnosis, ratio of total number of glycerin suppositories received to DOL of diagnosis, rate of hemodynamically significant patent ductus arteriosus (hsPDA), modality of hsPDA treatment, manipulation of hsPDA within 3 days of diagnosis, and hydrocortisone or dexamethasone exposure within 3 days of diagnosis (Table 4). hsPDA was defined as a PDA that necessitated medical or surgical treatment (2011–2017) or by Iowa PDA score (2018–2019) (41). Stool and glycerin ratios were obtained by counting the total number of stools passed and the total number of glycerin suppositories received prior to the day of diagnosis and dividing by the number of days of life at time of diagnosis.

**Table 4:** Risk factors for intestinal illness in infants diagnosed with NEC before and after initiation of probiotic supplementation in 2015.

	**Pre-probiotic epoch (2011–2015) *n* = 14**	**Post-probiotic epoch (2015–2020) *n* = 23**	**Significance**
Weight at diagnosis (g)	1,778 (±237)	1,687 (±216)	NS
DOL at diagnosis	15.4 (±2.3)	21.9 (±4.4)	NS
PMA at diagnosis	32.8 (±1.1)	32.5 (±1.1)	NS
Early onset sepsis (clinical + culture positive) (%)	5 (36)	15 (68)	*p* = 0.087
Late onset sepsis (clinical + culture positive) (%)	9 (64)	14 (64)	NS
Total stools/day of life at diagnosis	2.4 (±0.3)	1.7 (±0.4)	NS
Total glycerins given/day of life at diagnosis	0.07 (±0.03)	0.08 (±0.03)	NS
hsPDA (%)	3 (21)	12 (55)	*p* = 0.083
Method of hsPDA treatment			NS
Acetaminophen (%)	1 (33)	5 (28)	
Ibuprofen (%)	1 (33)	1 (6)	
Indomethacin (%)	0 (0)	7 (39)	
Ligation (%)	1 (33)	3 (17)	
Catheter closure (%)	0 (33)	2 (11)	
Hydrocortisone or dexamethasone within 3 days of diagnosis (%)	0 (0)	4 (19)	NS

*There were no significant differences between infants diagnosed with NEC in the post-probiotic epoch compared to the pre-probiotic epoch. There was a trend toward a higher rate of early onset sepsis and a higher percentage of infants with a diagnosis of hemodynamically significant patent ductus arteriosus (hsPDA) in the post-probiotic epoch compared to the pre-probiotic epoch, but these trends did not reach statistical significance*.

Risk factors for intestinal illness were overall similar between the infants with NEC in the pre-probiotic epoch compared to the post-probiotic epoch. There were non-significant trends toward a higher proportion of infants in the post-probiotic epoch compared to the pre-probiotic epoch who had clinical or culture positive EOS (68% vs. 36%) and who were diagnosed with a hsPDA (55% vs. 21%). However, modality of treatment and the proportion of infants who had manipulation of the hsPDA within 3 days of diagnosis of NEC was unchanged between the two groups. When analysis was restricted to inborn only infants, there was a significant increase in rate of diagnosis of hsPDA in the infants with NEC in the post-probiotic epoch compared to the pre-probiotic epoch (67% vs. 17%; *p* = 0.0361).

### Markers of Illness Severity

Data were collected regarding laboratory values (white blood cell [WBC] count, absolute neutrophil count [ANC], platelet count, and C-reactive protein [CRP]) on day of diagnosis and 1 day following diagnosis, need for inotrope or pressor therapy within 3 days of diagnosis, need for surgical management and type of surgical management if needed, total antibiotic days for the most common anti-microbial agents used in the first 30 days of life, and total days of total parenteral nutrition (TPN) for each case of NEC in the pre-probiotic and post-probiotic epochs (Table 5). Markers of illness severity were not significantly different between the infants with NEC in the pre-probiotic epoch and post-probiotic epoch.

**Table 5:** Markers of illness severity in infants diagnosed with NEC before and after initiation of probiotics of probiotic supplementation in 2015.

	**Pre-probiotic epoch (2011–2015) *n* = 14**	**Post-probiotic epoch (2015–2020) *n* = 23**	**Significance**
WBC count on day of diagnosis	12.5 (±1.8)	12.8 (±2.3)	NS
WBC count 1 day after diagnosis	9.9 (±1.5)	11.2 (±2.8)	NS
Platelet count on day of diagnosis	324 (±28)	320 (±45)	NS
Platelet count 1 day after diagnosis	301 (±43)	264 (±52)	NS
CRP on day of diagnosis	1.3 (±1.0)	2.9 (±1.1)	NS
CRP 1 day after diagnosis	5.1 (±2.2)	5.5 (±1.1)	NS
Surgical management			
Surgery required (%)	6 (43)	12 (55)	NS
Drain placement only (%)	0 (0)	0 (0)	NS
Exploratory laparotomy (%)	4 (29)	9 (41)	
Both drain + laparotomy (%)	2 (14)	3 (14)	
Bowel resection (%)	7 (50)	10 (45)	NS
Days receiving TPN prior to discharge	39.0 (±6.3)	44.0 (±8.0)	NS

*There were no significant differences in markers of illness severity in infants diagnosed with NEC in the post-probiotic epoch vs. in the pre-probiotic epoch*.

## Discussion

We performed a single-center retrospective cohort study examining rates of modified Bell's stage ≥2a NEC and all-cause pre-discharge mortality before and after initiation of routine supplementation of a multispecies probiotic supplementation in 2015. We found no difference in rates of NEC or all-cause mortality in VLBW infants in the post-probiotic epoch (2015–2019) compared to the pre-probiotic epoch (2011–2014), which was inconsistent with our initial hypothesis. When examining only ELBW infants, while there was a trend toward a higher rate of NEC in the post-probiotic epoch, this difference was not significant. Compared to infants diagnosed with NEC in the pre-probiotic epoch, infants with NEC in the post-probiotic epoch did have a modestly significant lower 5 min APGAR score (6.3 vs. 7.8; *p* = 0.0316) as well as an increased rate of probiotic use (65% vs. 0%; *p* < 0.0001). The current estimate of the incidence of NEC in VLBW infants is ~6–7% (2). This study makes it clear that the rate of NEC at our institution is well below average, as through consensus with our surgery colleagues, over a 9 year period, we have identified rates of NEC in VLBW infants of 0.7–3.9%. There are several possible factors that are contributing to our lack of efficacy for probiotic use to decrease our NEC incidence. First, NEC is a multifactorial disease and while probiotics have been shown to lower NEC rates, no study has taken NEC incidence to zero. Thus, it is possible that we did not see a decrease in the rate of NEC in the post-probiotic epoch because our institution may have a NEC rate lower than the threshold that can be impacted by probiotic use. Our overall average rate of NEC during both epochs combined (2011–2019) was 1.9% which is significantly below the national average and below the NEC rate in most probiotic publications (2, 42). Furthermore, if the rate of NEC in the year 2018 (3.9%) is removed as an outlier, NEC rates at our institution then range between 0.7 and 2.9%, with an overall NEC rate of 1.6% from 2011 to 2019. Other studies, some of which used the exact same formulation of probiotic that is used at our institution, have shown a significant decrease in NEC rate with probiotic use; however, the baseline rates of NEC in these studies prior to the intervention were appreciably higher than baseline NEC rates at our institution, ranging from 4.4–9.8% (22, 23, 25). While these studies found significant decreases in NEC following probiotic use, the post-probiotic NEC rate ranged from 2.0 to 5.4%, which is the approximate average rate of NEC at our institution regardless of probiotic use (22, 23, 25).

Secondly, our institutional feeding guidelines, especially in the ELBW population, favor routinely slow and cautious feeding volume advances, rarely exceeding increases of more than 10–15 mL/kg/day. As rapid feeding advancements are considered a risk factor for NEC, this may play a role in our low incidence. Lastly, we have extremely high rates of maternal and donor breast milk use, with subsequent low use of bovine-origin formula as the base feed which has been shown to be associated with lower rates of development of NEC (43, 44).

Interestingly, in the sub-group analysis of only ELBW infants, there was a non-significant trend toward higher rates of NEC in the post-probiotic epoch compared to the pre-probiotic epoch (4.1% vs. 1.6%). While this did not reach statistical significance, a 2.5-fold increase may be of clinical significance. However, it is important to note that there was an increase in practice heterogeneity during this epoch as we had a large turnover of both neonatologists and nurses during this time frame, which were unrelated to the initiation of probiotic supplementation, but may have confounded our results.

Following our institution's initiation of routine probiotic supplementation, 65% of the infants who went on to develop NEC received probiotics at some point in their course, an increase from the pre-probiotic epoch (0%). There were several cases of NEC in term infants in the post-probiotic epoch, and since these infants do not receive probiotic supplementation routinely at our institution, the rate of probiotic use in the NEC population was lower than expected. When we only examined VLBW infants with NEC in the post-probiotic epoch, we found that the rate of probiotic use increased to 94%, and when we further restricted this analysis to the ELBW population, who are at highest risk for development of NEC, we found that the rate of probiotic use was 93%. There was a single infant who fell into both the VLBW and ELBW categories who did not receive probiotics. This infant was outborn and developed NEC at the hospital of birth; following transport to our facility the infant was critically unwell and passed away several weeks later due to complications from NEC and was never stable enough to receive probiotic supplementation. The rate of probiotic use was even more pronounced when analysis was restricted to inborn only VLBW infants, all of whom (100%) received probiotic supplementation at an average of 7 days of life.

The analysis of our data found a modestly significant lower average 5-min APGAR score in the infants with NEC in post-probiotic epoch compared to the pre-probiotic epoch (6 vs. 8). While a drop in APGAR score from 8 to 6 may be interpreted by some providers as substantial, we believe this is unlikely to be clinically significant. First, although many studies have attempted to link APGAR scores with long-term metrics, these studies were both unsuccessful and attempted to causally link APGAR scores with a long-term deficit such as lower intelligence or neurodevelopmental impairment (45). Second, there is significant inter-user variability in assigning APGAR scores (46) and this variability in and of itself may at least partially explain the difference we saw between the two groups. Third, although a lower 5 min APGAR score could indicate that the infants with NEC in the post-probiotic epoch were more ill at baseline, and therefore possibly at increased risk for complications such as NEC, we examined multiple other metrics of illness severity (Table 5) and found no significant differences between the infants with NEC in the two epochs. Similarly, the lower APGAR score could also be related to the non-significant trend toward a higher percentage of outborn infants who went on to develop NEC in the post-probiotic epoch compared to the pre-probiotic epoch (48% vs. 14%), since resuscitation of high risk and critically ill infants is often more difficult in lower-resource settings. Finally, APGAR scores are assigned in the minutes after an infant is born and therefore would not have been affected by later supplementation with probiotics, which did not occur until an average of DOL 7 for inborn infants and DOL 28 for outborn infants. There was also a trend toward a higher rate of EOS in infants with NEC in the post-probiotic epoch compared to the pre-probiotic epoch, which again may indicate that the infants with NEC in the post-probiotic epoch were more ill at baseline, but none of these cases were culture positive, and this did not reach statistical significance, making its actual effect on our study unclear.

Lastly, we noted a higher proportion of infants with NEC in the post-probiotic epoch compared to the pre-probiotic epoch who were diagnosed with hsPDA (55% vs. 21%). This finding is most likely due to diagnostic bias. In 2018, during the post-probiotic epoch, our institution implemented a neonatologist-performed targeted neonatal echocardiography (TnECHO) protocol for screening all infants born <27 weeks for hsPDA in the first 18–24 h of life. Prior to this, echocardiograms were only obtained in the postnatal period if there was clinical suspicion for hsPDA as opposed to universal screening. Because of this universal screening TnECHO protocol for hsPDA in premature infants at our institution, more cases of hsPDA were likely discovered and treated, contributing to this trend. When we restricted our analysis to inborn only infants, who were subject to this protocol, the proportion of infants with hsPDA who developed NEC was significantly higher in the post-probiotic epoch compared to the pre-probiotic epoch, confirming that these findings are likely due to diagnostic bias.

This study has several limitations. First, this study is retrospective in nature and prone to many confounding variables and biases for which we were not able to completely control. There continues to be considerable controversy regarding probiotic supplementation in preterm infants, and retrospective data is not nearly as convincing or definitive as randomized trials and prospective studies. Although some small single center trials and prospective studies involving probiotic supplementation have been successfully completed showing a positive benefit to risk ratio, the collective data is still mixed on whether probiotics provide definitive benefit to premature infants and currently the AAP does not recommend universal probiotic supplementation to premature infants (31). Another limitation of this study is that it only includes data from a single center where approximately one third of infants are outborn and spend various periods of time at lower level NICUs prior to transfer to our institution, where there is substantial variation in clinical care from what we provide. However, as discussed above, even when analysis was restricted to inborn only infants, there still was not a significant reduction in our rate of NEC in the post-probiotic.

In conclusion, this study demonstrates that at our institution, routine supplementation of a multispecies probiotic had no effect on rates of modified Bell's stage ≥2a NEC or all-cause mortality. This contrasts with multiple other studies in the literature, some using the exact same probiotic strain, where there were significant reductions in these metrics. We hypothesize that these findings were likely due to a low baseline rate of NEC at our institution which fell below the threshold of probiotic efficacy. Additional large scale randomized control trials and prospective studies, in addition to improved standardization and regulation of probiotic strains by the FDA, are needed in order to definitively determine whether probiotics provide a conclusive benefit and importantly which strains and preparations are the most effective in reducing NEC and mortality in preterm infants.

## Data Availability Statement

The raw data supporting the conclusions of this article will be made available by the authors, without undue reservation.

## Ethics Statement

The studies involving human participants were reviewed and approved by University of Iowa Institutional Review Board (IRB#201410743). Written informed consent from the participants' legal guardian/next of kin was not required to participate in this study in accordance with the national legislation and the institutional requirements.

## Author Contributions

BJ and SM: conceptualization, manuscript preparation (original draft), statistical analysis, and funding. BJ, TB, and SM: methodology. BJ and TB: data collection. BJ, TB, GP, and SM: consensus on NEC cases and manuscript preparation (review). SM: supervision and project administration. All authors contributed to the article and approved the submitted version.

## Funding

BJ was supported by grant funding NIH/NIAID T32AI007260. SM was supported by the Stead Family Department of Pediatrics.

## Conflict of Interest

The authors declare that the research was conducted in the absence of any commercial or financial relationships that could be construed as a potential conflict of interest.

## Publisher's Note

All claims expressed in this article are solely those of the authors and do not necessarily represent those of their affiliated organizations, or those of the publisher, the editors and the reviewers. Any product that may be evaluated in this article, or claim that may be made by its manufacturer, is not guaranteed or endorsed by the publisher.
